# Association between Asthma and Type 2 Diabetes Mellitus: Mechanisms and Impact on Asthma Control—A Literature Review

**DOI:** 10.1155/2021/8830439

**Published:** 2021-01-13

**Authors:** Raimeyre Marques Torres, Marcela Dos Santos Souza, Ana Carla Carvalho Coelho, Luane Marques de Mello, Carolina Souza-Machado

**Affiliations:** ^1^Graduate Program of the School of Nursing at the Federal University of Bahia, Salvador (BA), Brazil; ^2^Department of Social Medicine, School of Medicine, University of São Paulo, Ribeirão Preto (SP), Brazil

## Abstract

The study aimed to analyze the scientific production on the association between asthma and type 2 diabetes mellitus (T2DM) in adults, the mechanisms that explain this association, and its impact on asthma control. A literature review of scientific articles indexed in the MEDLINE/PUBMED, BVS, CINAHL, Cochrane Library, and Web of Science databases was carried out, considering publications from January 2009 to December 2019, using the following descriptors: “asthma”, “type 2 diabetes”, “adult,” and “association”. Of 962 articles found, 18 were included because they met the eligibility criteria. It is suggested that the association between asthma and T2DM is caused by low-grade systemic inflammation (7 articles) or the use of corticosteroids (7 articles). It is noticed that there is a limited scientific production regarding the consequences of this association for the control of asthma (5 articles). It is concluded that asthma and T2DM are two common chronic conditions of increasing prevalence and that often coexist in the same patient. It is suggested that this coexistence worsens asthma control. Therefore, the study may support public policies and clinical health practices that value the approach of comorbidities associated with asthma such as T2DM, in order to minimize additional health risks and reduce the quality of life.

## 1. Introduction

Asthma is a heterogeneous disease, characterized by chronic inflammation and airway hyperresponsiveness [1]. Type 2 diabetes mellitus (T2DM) is a multifactorial disease, mainly characterized by a decrease in insulin sensitivity and also by a defect in insulin secretion and chronic inflammation [2].

The notion of asthma as a primary pulmonary disease has been discussed [3], but studies show that asthma is associated with extrapulmonary comorbidities such as obstructive sleep apnea syndrome (OSAS) [4] and systemic diseases such as obesity [5], diabetes mellitus (DM) [6], and metabolic syndrome (SM) [7].

Asthma, obesity, and T2DM are very common chronic health conditions [5, 8]. Currently, estimates reveal 339 million asthmatics, 463 million individuals with DM, and 650 million obese worldwide [8–10]. In Brazil, data show a general prevalence of 6.4% of individuals with asthma, 9.4% with DM, and 19.8% with obesity among adults [9, 11, 12], resulting in an economic burden associated with morbidity and considerable mortality [1, 9]. The prevalence of those chronic conditions in developing countries is increasing rapidly, probably as a result of the westernization of lifestyle [9, 13].

Obesity seems to be an important contributor to the association asthma and T2DM, but the mechanism linking those three chronic conditions is poorly understood [3, 5]. Asthma can interact synergistically with obesity, increasing circulating levels of inflammatory cytokines leading to an increased risk of insulin resistance and T2DM [14]. However, asthma has been associated with an increased risk of T2DM in women, regardless of body mass index (BMI), indicating that chronic inflammation contributes to the pathogenesis of diabetes [15]. The role of adiposity in pathogenic pathways linking asthma and T2DM needs to be further studied [16, 17].

Comorbidities are important factors concerning the management and prognosis of asthma, as they are associated with inadequate disease control, greater use of health care, and poorer quality of life [18]. Studies suggest that the presence of comorbidities such as T2DM is more common in individuals with asthma than in controls without asthma [6, 14, 15, 18, 19] and may be associated with a worse outcome in asthma, contributing to greater use of health resources and worse quality of life [18, 20, 21].

The possible mechanisms involved in the association between asthma and chronic diseases are not yet well known, but some hypotheses have been discussed. More recent studies have shown that several factors may explain this association, such as low-grade systemic inflammation [14, 15], genetic pleiotropy [22], oxidative stress [14], and use of corticosteroids [23–25]. It is important to note that the various plausible mechanisms for the increased risk of T2DM among asthmatics can be altered or aggravated by the asthma phenotype [14].

Taking these aspects into consideration, this study aims to analyze the scientific production on the association between asthma and T2DM, the mechanisms that explain this association, and its impact on asthma control. This theme is relevant, as it may contribute to the development of public policies and clinical practices that guarantee the improvement of care for people with coexisting chronic conditions such as asthma and T2DM, aiming at a comprehensive approach at all healthcare levels for these individuals, as well as better quality of life and lower costs.

## 2. Methods

This is a literature review of observational and intervention studies that investigated the association between asthma and T2DM. The search for the articles was performed using the electronic databases Medical Literature Analysis and Retrieval System Online (MEDLINE/PUBMED), Virtual Health Library (VHL), Cumulative Index to Nursing and Allied Health Literature (CINAHL), Cochrane Library, and Web of Science. We also conducted a manual screening of reference lists of articles, reviews, and other documents, to identify additional studies potentially eligible for the review. There was no restriction on the language of the article.

After analyzing the terms identified in the Health Sciences Descriptors (DECS) and Medical Subject Headings (MESH), the following descriptors were identified: asthma (*asma*), type 2 diabetes mellitus (*diabetes mellitus tipo 2*), adult (*adulto*), and association (*associação*). To design the search strings, the “AND” Boolean connector was used to combine the search terms, namely, (i) asthma AND type 2 diabetes mellitus (*asma* AND *diabetes mellitus tipo 2*); (ii) asthma AND diabetes mellitus type 2 AND adult (*asma* AND *diabetes mellitus tipo 2* AND *adulto*); (iii) asthma AND type 2 diabetes AND adult AND association (*asma* AND *diabetes mellitus tipo 2* AND *adulto* AND *associação*).

The review of the selected articles was carried out by two authors: A1 and A2. A1 collected the data from the online databases, and A2 reviewed them. A1 and A2 discussed the results for the homogeneity of the information according to the pre-established criteria. After excluding duplicate articles, articles that met the inclusion and exclusion criteria were read and fully analyzed. Original articles were included, with adult participants, and published from January 2009 to August 2019, taking into account that, during this period, the association asthma and T2DM started to arouse the interest of the scientific community, as it is a concomitance between two chronic conditions prevalent in the general population and of great epidemiological impact. In vivo animal studies, in vitro/ex vivo investigations, review studies, meta-analysis, theses, conference abstracts, letters to the editor, and full texts not available were excluded.

There was also the participation of a third author (A3) in case of disagreement on the topics discussed in the article. The final version of the discussions and results was also analyzed and reviewed. The association between asthma and T2DM was defined as the primary outcome and secondary outcomes were: mechanisms that explain the association between asthma and T2DM and the impact of this association for the control of asthma and T2DM.

The evaluation and structuring of this study followed the criteria of the Preferred Reporting Items for Systematic Reviews and Meta-Analyses (PRISMA) [26]. It should be noted that the PRISMA items referring to meta-analysis (12–16 and 19–23) were not used because it is a literature review.

## 3. Results

**Figure 1:**
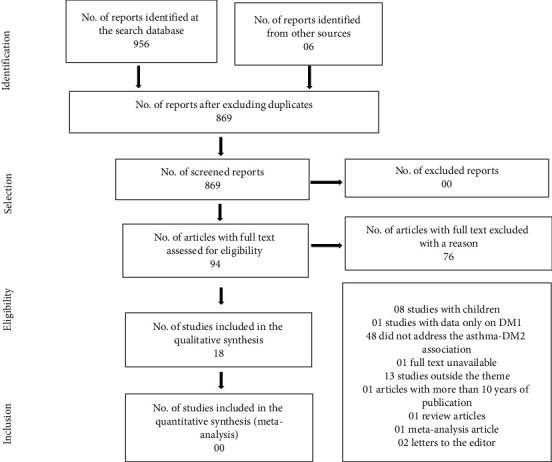
Flowchart of articles included and excluded in the review, 2019.

A total of 956 articles were identified at the databases and 6 articles from the references of the selected articles that met the inclusion criteria. After excluding duplicates and reading the titles and abstracts, 94 articles were potentially eligible and selected for full reading. Seventy-six articles were excluded, resulting in a total of 18 articles selected according to the eligibility criteria (Figure 1). The flowchart presents the process of selecting studies according to the items of PRISMA [26].

All eighteen articles selected described the association between asthma and T2DM. Seven studies advocated low-grade systemic inflammation as a mechanism that associates asthma and T2DM [14–16, 19, 28, 30–31], 7 studies the use of medications as factors that associate asthma with DM2 [19, 23, 27, 32–34], and 5 studies addressed the impact of the association between asthma and T2DM for asthma control [20, 21, 37–40]. One study had data available for low-grade systemic inflammation and medication use as mechanisms that associate asthma with T2DM; thus this article was included in both subanalyses [19]. The other characteristics related to the study site, sample characteristics, and results related to the analysis of the association between asthma and T2DM are described in Table 1.

## 4. Discussion

### 4.1. Association between Asthma and T2DM: Epidemiological Aspects

All eighteen articles selected described the association between asthma and T2DM. However, there was a potential bidirectional relationship between these two chronic conditions. In the Singapore Chinese Health Study [14], the Women's Health Study [15], the SHIELD study [26], and the population study among residents in Rochester, Minnesota [19], the authors examined the incidence of diabetes in those with asthma. In the study with the population of twins who were in the Danish Twin Registry [28] and in the study using data from veterans' hospital discharge [29], the authors reported rates of prevalence or incidence of asthma in people with T2DM. However, a longitudinal observational study found a negative association in the concomitance between asthma and T2DM [16].

The epidemiological impact of the association between asthma and T2DM is still poorly described. Longitudinal observational studies have found a risk of between 21% and 37% of the incidence of T2DM in adults with self-reported asthma [14, 15, 19]. Thomsen et al. observed an almost doubled asthma risk in patients with T2DM compared to individuals without T2DM in a large study among Danish twins [28]. Using administrative data from veterans' hospitals, Hashemzadeh and Movahed [29] found that asthma was present in 4.5% of patients with T2DM vs. 2.9% in the control group, regardless of other comorbid conditions.

From the review of the selected articles (Table 1), it was possible to observe that asthma and T2DM are two common chronic conditions of increasing prevalence [1, 9] and that frequently coexist in the same patient [14, 15, 28]. It is hypothesized that low-grade systemic inflammation [14–16, 19, 28, 30, 31] and the use of medications, particularly corticosteroids [19, 23, 27, 29, 32–34], may be the cause of this association. Hyperglycemia and hyperinsulinemia in the lungs also appear to contribute to reduced lung function and a higher occurrence of asthma in patients with diabetes [35, 36]. It should also be noted that the findings of studies compiled in this review point to worse asthma control in individuals with asthma and T2DM, increasing the chances of exacerbations, complications, and hospitalizations [20, 21, 37–40].

### 4.2. Mechanisms that Explain the Association between Asthma and DM2: Low-Grade Systemic Inflammation

In seven studies, the mechanisms explaining the association between asthma and T2DM were presented, from the perspective of low-grade systemic inflammation [14–16, 19, 28, 30, 31]. Scientific evidence proves that asthma [40] and T2DM [14, 41, 42] are associated with low-grade systemic inflammation, dependent or independent of obesity.

In the prospective cohort study carried out by Mueller et al. among Chinese Singaporeans followed for 5.7 years, there was a significant association between asthma and the incidence of T2DM diagnosed by a doctor. This association appeared to be stronger for obese participants compared to that for nonobese participants, suggesting that excess body fat and airway inflammation may interact to promote the development of diabetes [14].

A similar study was carried out by Thomsen et al. with a population of Danish twins, showing an increased risk of asthma in individuals with T2DM compared to nondiabetic individuals, for both sexes. Within this analysis, significantly positive genetic correlations between asthma and BMI were also found in women, suggesting a common etiology for asthma and elements of the metabolic syndrome such as low-grade systemic inflammation [28].

Song et al., studying female health professionals with pre-existing asthma for an average period of 12 years, also found an increased risk in the incidence of T2DM when compared to the control group. The authors concluded that asthma was individually and independently associated with an increased risk of T2DM, indicating that chronic inflammation contributes to the pathogenesis of diabetes [15]. Corroborating these findings, a study developed by Gulcan et al. has shown that chronic inflammation that permeates asthma increases insulin resistance and the risk of T2DM. This inflammation was characterized by increased levels of ultrasensitive C-reactive protein (CRP-us), LDL-C, and the number of leukocytes in the asthmatic group, compared to those in the control group [18].

In a retrospective cohort study developed by Yun et al., participants with asthma and nonasthmatic controls were compared regarding the risk of developing proinflammatory conditions such as coronary heart disease (CHD), T2DM, inflammatory bowel disease (IBD), and rheumatoid arthritis (RA). The authors found an increased risk of T2DM (HR = 2.12 (95% CI 1.43–3.14), *p* value> 0.001), and CHD (HR = 1.50 (95% CI 1.07–2.10), *p* value 0.02) in asthma and argue that the association with asthma-T2DM and asthma-CHD probably involves the sharing of common immunogenic and environmental conditions, such as production of inflammatory cytokines IL-6 and IL-17 already identified in all those clinical conditions and extrinsic mechanisms such as hypoxia and tachyarrhythmia present in exacerbations of asthma and capable of triggering symptoms of CHD. They also discuss the use of corticosteroids revealing those with an immunogenic predisposition to T2DM. However, the authors did not evaluate BMI as a possible confounding factor, which may have influenced the observed results [19].

A study by Verbovoy et al. discussed also the presence of subclinical inflammation characterized by hyperleptinemia, hyperresistinemia, lower adiponectin levels, and increased levels of IL-6 and IL-10 in women with T2DM and bronchial asthma when compared to the control group. In those women, the indicative of obesity was BMI and waist circumference (WC) characteristics of visceral obesity, in addition to positive correlation between the concentration of leptin and WC, between the levels of resistin and insulin and the levels of resistin and HOMA [31].

Another study, carried out by Baek et al. in order to verify the influence of T2DM on the incident risk of asthma, using data from the Korean National Health Insurance Service/NHIS, concluded that T2DM and asthma share common pathophysiology-chronic inflammation. However, the results of the study showed that patients with T2DM without retinopathy had a lower risk of developing asthma in nondiabetic individuals, even after adjusting for confounding covariates such as BMI, arterial hypertension, and dyslipidemia. On the other hand, the diabetic individual with retinopathy had a higher risk of developing asthma, suggesting that the vascular network of the lungs may be the target of T2DM microvascular complications [16].

Obesity is a chronic condition that leads to systemic inflammation and, when present, has been identified as the main explanation for the association between asthma and T2DM. However, although excess body fat and airway inflammation in asthma can interact to promote the development of diabetes [14, 31], it also shares the common inflammatory bases with T2DM, regardless of the increase in BMI [15, 30].

A number of hypotheses have been proposed to explain the biological mechanisms underlying the concomitance of obesity with asthma [5] and its association with T2DM [14, 22, 28]. Obesity is associated with low-grade systemic inflammation, characterized by increased levels of inflammatory markers such as the Toll-like receptor 4 (TLR4), tumor necrosis factor-alpha (TNF-*α*), IL-6, PCR, and IL -1*β*, capable of promoting changes in the immune system response or in the inflammatory response [22, 35, 41, 43], which can be the basis of the process that promotes metabolic and immunological dysregulation, increasing the risk for other inflammatory conditions such as atherosclerosis, diabetes, and cardiovascular disease [22, 41, 43, 44].

In particular, the inflammatory pathway of TNF-*α* and IL-6 appears to be the link between those three chronic conditions [35]. TNF-*α* and IL-6 favor the differentiation of Th2 cells and the production of cytokines such as IL-4 and IL-5 involved in the typical inflammation observed in eosinophilic asthma [13, 35]. Those cytokines are also capable of inducing the differentiation of Th2 cells into Th17, and a microenvironment rich in Th17 cells and elevated levels of IL-17 can be seen in obese target people and those with T2DM [35] and severe neutrophilic asthma [45]. In addition, TNF-*α* increases circulating levels of leptin and resistin when interacting synergistically with IL-6, favoring increased peripheral insulin resistance [2, 32, 45–47] and greater chances of developing T2DM [22, 48].

Inflammatory signaling pathways can also be triggered by the activation of toll-like receptors (TLRs), affecting the modulation of the innate immune response and are expressed in target insulin tissues, such as the liver, adipose tissue, and pancreatic cells [22]. The activation of TLR4 by free fatty acids, elevated in obesity, generates proinflammatory signals and activation of NF-*κ*B that inhibits the transmission of the insulin signal [2]. It has also been emphasized that obesity causes metabolic tensions within the cell, leading to stress in the endoplasmic reticulum and overproduction of reactive oxygen species, causing damage to mitochondrial components and mitochondrial loss, and contributing to insulin resistance and causing greater activation of inflammatory cells [49].

Obesity causes changes in the circulating levels of adiponectin, leptin, and resistin by adipocytes, which can act as a regulatory link between the endocrine system and the immune system, playing a role in the relationship between obesity, asthma, and diabetes [22, 41, 46, 50]. Leptin induces the expression of TNF-*α*, IL-6, and IL-1*β* in adipose tissue cells, contributing to insulin resistance, and is considered one of the links between obesity, insulin resistance, and atherosclerosis [43, 44]. In contrast, adiponectin reduces the lipid storage capacity of adipocytes, inhibits liver neoglucogenesis, and increases the sensitivity of cells to the action of insulin [22, 44]. Hyperleptinemia, hyperresistinemia, and lower concentrations of adiponectin, as well as elevated levels of IL-6 and reduced levels of IL-10, were found in patients with T2DM and in those with concomitant T2DM and asthma [31].

In addition, systemic inflammation in obesity, particularly abdominal obesity (high waist circumference), is an important parameter to define other metabolic conditions such as metabolic syndrome (MS) that can be a link between asthma and T2DM. A study proves the association of obesity, MS, and airway diseases [49]. The Norwegian prospective cohort reported that MS was a risk factor for the incidence of asthma (OR 1.6), mainly two of its components, waist circumference (OR 1.6), and high blood glucose (OR 1.4), even after mutual adjustment for other metabolic components [51]. A study among Indian adults aged 17 to 59 years suggests that patients with obesity and MS may be a subtype of asthmatics with more severe asthma (*p* < 0.05), worse quality of life (*p* < 0.05), high risk of apnea obstructive sleep (*p* < 0.05), and higher levels of inflammatory markers (leptin and IL-6, *p* < 0.05) compared to nonobese asthmatic patients and obese asthmatics without MS [47].

However, despite strong evidence that the association of asthma with other systemic comorbidities can count on the participation of inflammation typical of obesity, a prospective study with children demonstrated that asthma is directly associated with dyslipidemia, insulin resistance, and hyperinsulinemia, known precursors of cardiovascular disease, and diabetes, even in the absence of obesity [52]. A similar result was found in a large Danish cohort, where it was observed that insulin resistance was more strongly related to the risk of asthma in adults than obesity [48]. The evident inflammation of the adipose tissue, insulin resistance, hepatic steatosis, and dyslipidemia and frequent changes in obesity were also found in a condition with reduced fat mass, such as lipodystrophy [2].

Hyperglycemia and hyperinsulinemia are characteristics of insulin resistance and are present in MS and T2DM (treated and untreated) [35]. The high levels of glucose or insulin in the lungs promote the proliferation and differentiation of fibroblasts, resulting in collagen deposition and airway remodeling, contributing to the reduction of lung function in patients with diabetes [35, 36]. Alterations in the alveolar capillaries and pulmonary arterioles, chronic low-grade inflammation, autonomic neuropathy, and elastic loss secondary to the glycosylation of the pulmonary parenchyma collagen are also other mechanisms pointed out as responsible for the decreased pulmonary function observed in patients with diabetes mellitus [53–55].

Data from a systematic review of cross-sectional studies showed that adults with diabetes (without asthma), when compared to their peers without diabetes, had lung volumes 3 to 10% lower (FVC more consistent than FEV1), regardless of BMI and smoking; the findings in longitudinal studies are less consistent with some studies that identified an increased rate of decline in lung function in adults with diabetes and other studies that reported an association between decreased lung function (mainly FVC), insulin resistance, and diabetes incidence [53]. Similar findings have been reported among rural adults in Australia [55] and among Hispanics/Latinos in the United States [21]. In this last study, the impairment of lung function was greater, especially in forced vital capacity (FVC), among participants with T2DM and lung disease (asthma, chronic bronchitis, and emphysema).

Insulin resistance appears to be a strong and independent risk factor for the development of asthma, but how insulin acts in the lungs and its local effects is not yet clear [36]. Data from clinical trials to test new insulin presentations, such as inhalers, provided interesting information [35]. In an experimental study, intranasal insulin administration induced insulin resistance, increased levels of catenin, collagen deposition, and bronchial hyperresponsiveness. Catenin has been identified as a factor significantly associated with insulin-induced changes in lung function [35]. However, it is unknown whether insulin resistance is a causal factor in the development of asthma or simply the result of the action of inflammatory mediators also involved in the pathogenesis of asthma [22, 48].

### 4.3. Mechanisms that Explain the Association between Asthma and DM2: Drug Effects

Seven studies retrieved from the bibliographic search explain the association between asthma and DM2 as a result of the use of medications to control asthma, mainly the use of corticosteroids, both systemic and inhaled [19, 23, 27, 29, 32–34]. Some authors point out that the use of systemic corticosteroids is associated with several adverse metabolic effects, including insulin resistance and glucose intolerance, as well as hyperglycemia and DM [24]. Other authors report that the use of inhaled corticosteroids (IC), especially at higher doses, is related to small disturbances in glucose control [46, 47]. However, glycemic changes due to the use of IC are still controversial [56].

Two population-based studies [19, 27] identified diabetes as a comorbidity in asthma. In the first study, the diagnosis of asthma was associated with a 2.11 times increased risk of developing DM (95% CI (1.43–3.13), *p* < 0.001) [19]. In the second study, it was observed that adults with asthma had a significantly increased risk (33%) of developing T2DM (*p*=0.020) [27]. For these authors, the presence of diabetes as a comorbidity in asthma may be associated with the use of glucocorticoids, which may reveal the predisposition to diabetes.

Similar results were found in two studies conducted in patients after hospitalization for exacerbation of asthma. In the study by Koskela et al., patients with asthma without a diagnosis of diabetes who used corticosteroids evolved with hyperglycemia [32]. A study carried out with patients in the archives of military hospitals also showed that asthmatic patients undergoing treatment with corticosteroids developed T2DM when compared to the control group, even after adjusting for possible confounders such as hyperlipidemia and smoking [29]. For those authors, the association between asthma and diabetes was the result of treatment with systemic corticosteroids and their effects on glucose metabolism.

Corroborating those findings, the first study carried out to determine the prevalence of morbidities associated with severe asthma carried out in two large databases in the United Kingdom found that systemic exposure to steroids induces T2DM [23]. Results of the study by Suissa et al. permeate the aforementioned findings; however, the use of IC was associated with an increased risk of diabetes incidence [33]. That study also showed that increasing the dose of IC leads to an increase in the risk of T2DM and the risk of progression to insulin therapy.

A pioneer prospective study among diabetic patients with concomitant asthma or COPD carried out in an outpatient clinic for veterans in the United States agrees with the findings cited above, in demonstrating that the mean difference in glycated hemoglobin (HbA1c) from baseline in 6 weeks was significantly greater after treatment with fluticasone propionate (FP) than after therapy with oral montelukast (*p* < 0.05) [34]. Those results demonstrate that the moderately high dose of corticosteroids for the treatment of asthma or COPD is associated with minor disturbances in glucose control after a short follow-up period. The changes are detectable but smaller than those that would normally be considered clinically relevant and are not sufficient to suspend the therapy in use. However, the authors suggest that clinicians carefully monitor blood glucose control in patients who initiate IC, especially at higher doses.

The studies included in this category have shown that insulin resistance, hyperglycemia, and diabetes are more common than expected in patients with asthma exacerbation, especially when systemic corticosteroids were used, revealing those individuals with an immunogenic predisposition to DM [19, 23, 27, 29, 32]. Some authors attribute less participation of corticosteroids in this process, arguing that, today, systemic corticosteroids are less used and IC is not associated with frequent glycemic changes [56]. The literature recommends the preferential use of IC, even at higher doses, thus being possible to reduce the chances of glycemic lack of control, since the use of systemic corticosteroids may result in hyperglycemia or the deterioration of glycemic control, requiring progression to insulin therapy [32–34, 55].

Exposure to systemic glucocorticoids has several side effects, including a diabetogenic action that occurs through different mechanisms: (1) increases hepatic gluconeogenesis; (2) decreases the peripheral glucose uptake in the skeletal muscle, liver, bone, and adipose tissue; (3) decreases the insulin sensitivity and antagonizes its effect; (4) inhibits the insulin secretion by *β* cells; and, (5) favors the systemic release of fatty acids and triglycerides with the consequent lipotoxicity that negatively affects the function of *β* cells. As a final result, glucocorticoids worsen the metabolic control of patients with diabetes, are responsible for the occurrence of hyperglycemia in nondiabetic individuals, and contribute to insulin resistance, glucose intolerance, hyperglycemia, and diabetes in individuals susceptible to diabetes [24, 56–58].

There are several risk factors for the development of hyperglycemia in individuals using systemic glucocorticosteroids, including older age, higher fasting plasma glucose levels and HbA1c, lower estimated glomerular filtration rate, pregnancy, visceral obesity, insulin resistance, and first-degree relatives of patients with diabetes [57, 58]. However, a study conducted with inpatients with rheumatic or kidney disease who developed diabetes due to the use of glucocorticosteroids found no significant differences between the two groups regarding the maximum or total dose of glucocorticoids [57].

Considering that inhaled corticosteroids are the basis of asthma therapy, a review study carried out to assess the systemic effects of those medications concluded that they have an excellent safety profile when administered in low doses and that the risk of developing diabetes and progression for insulin therapy is dose-dependent [25]. Another study conducted with a cohort of American veterans, with and without diabetes, identified by self-report or by the use of hypoglycemic agents, showed that the use of IC by individuals with diabetes was associated with increased dose-dependent concentrations of serum glucose [59]. However, a study that evaluated results of double-blind, placebo-controlled clinical trials in patients ≥4 years of age with asthma using budesonide did not identify an increased risk of new diabetes or hyperglycemia associated with the use of IC regardless of the dose used [56].

In severe asthma and other nonrespiratory pathological conditions, the use of systemic corticosteroids is still a reality and may contribute to the increase of adverse metabolic effects, including insulin resistance, hyperglycemia, and T2DM [23, 24]. Data from two major British bases portrayed in the pioneering study conducted by Sweeney et al., concluded that comorbidities associated with systemic exposure to corticosteroids are more common in severe asthma, including T2DM. For those authors, compared to mild to moderate asthma, rates of T2DM in severe asthma were significantly higher for conditions associated with systemic exposure to corticosteroids (severe asthma 10% vs. 7% in mild to moderate asthma, *p*=0.006). T2DM was also more common in individuals with severe asthma who required daily treatment with systemic corticosteroids to maintain asthma control than in individuals with severe asthma who had only frequent rescue cycles with the use of oral corticosteroids [23].

Taking into account that the use of systemic corticosteroids may be related to the increase in comorbidities in asthma such as T2DM, the search for new effective and safe treatments for this public is justified. In recent years, several drugs in the biological class have been developed, and omalizumab, a humanized anti-IgE monoclonal antibody, was the first to be used in the treatment of asthma [60]. However, those drugs also have adverse effects such as changes in glucose homeostasis [60, 61]. Two studies suggest an increase in blood glucose and the need for extra doses of insulin after starting treatment with omalizumab, and although the mechanism responsible for this change is not well understood, it is believed to be due to an increase in the histamine-induced release of biological or sucrose contained in the bottle of this medication [61, 62].

### 4.4. Impact of the Association between Asthma and DM2 for Asthma Control

Glycemic changes [20], diagnosis of long-standing diabetes [39], diabetic microangiopathy [20], and inflammation [37, 38] were identified as important characteristics for the loss of asthma control when associated with T2DM from reading the 5 studies included in the analysis of this category.

A prospective study by Wytrychowski et al. demonstrated that the hyperglycemia that accompanies systemic treatment with corticosteroids hinders recovering from the acute exacerbation of asthma that requires hospitalization and prolonging hospital stay compared to patients who do not have glucose metabolism disorders. The authors conclude that hyperglycemia is a factor that significantly increases the length of hospital stay for exacerbation of asthma, regardless of the mode of insulin therapy [21].

Another study, evaluating diabetic patients and nondiabetic controls who were hospitalized for acute asthma exacerbation (72%) or COPD (28%), showed that an exacerbation of prediagnosed diabetes contributed 3 times more to long-term mortality in individuals with asthma and concomitant obstructive diseases than glycemic changes during hospitalization. The authors argue that a history of diabetes, but not hyperglycemia during the exacerbation of the obstructive pulmonary disease, has an impact on long-term mortality but does not make explicit the pathophysiology involved with the outcome of this study [39].

Confirming those findings, a multicenter and observational study with cross-sectional analysis showed that Hispanics/Latinos aged 18 to 74 years with pulmonary disease (asthma, bronchitis, and COPD) and T2DM had mean values of forced expiratory volume in the first second (FEV1), lower forced vital capacity (FVC), and higher mean dyspnea scores than those without T2DM, regardless of age, sex, and smoking. The authors conclude that intrinsic biological changes in the lung related to diabetic microangiopathy cause pulmonary symptoms and dysfunction, which may contribute to general morbidity and mortality from lung diseases [21].

Veryomenko and Beztitko, studying the patients with moderately severe uncontrolled asthma and concomitant DM2, proved that the decrease in inflammatory markers and BMI reduced HbAlc and the level of glycemic load in the intervention group, improving asthma control by the Asthma Control Questionnaire by 21% [37]. A similar result was found by Li et al. when analyzing the anti-inflammatory effects of metformin in asthma control. The findings of this study confirm that the reduction in airway inflammation, provided by the use of metformin, contributed to a lower risk of exacerbation and hospitalizations for asthma when associated with T2DM [38].

Studies that assess the impacts of the association of asthma and T2DM on asthma control are still scarce. However, it is hypothesized that the combination of asthma and T2DM can compromise glycemic control both in the short term, contributing to an unfavorable effect in the course of exacerbation of asthma and duration of hospitalization [16, 21], and in the long term, when the history of diabetes and the presence of pulmonary microangiopathy increase the risk of late deaths [21, 39]. In fact, a study proves that hyperglycemia during the exacerbation of chronic obstructive pulmonary disease predisposes to a longer average hospital stay [63]. On the other hand, current results suggest that extensive microvascular complications, especially in adults with advanced diabetes, contribute to increased mortality from respiratory problems in adults with diabetes [21].

Possible explanations for the lack of glycemic control in the coexistence between asthma and T2DM are hypothesized, such as the presence of low-grade systemic inflammation and the use of corticosteroids. Inflammation is a significant cause of decreased insulin sensitivity and a critical driving force in complications associated with T2DM [2, 64]. A cross-sectional study with young diabetic people designed by Black et al. concluded that the inflammatory process underlying the asthma association and diabetes, permeated by obesity, may contribute to low glycemic control, especially in asthma exacerbation [65]. On the other hand, studies conclude that loss of glycemic control in individuals with asthma and diabetes may be associated with an intrinsic mechanism secondary to the use of systemic corticosteroids [19, 23, 24].

Hyperglycemia generates glycosylated proteins that interact with a specific receptor present in all cells, inducing oxidative stress and proinflammatory responses, leading to microangiopathic complications [54]. Microvascular structures such as the alveolar-capillary network in the lung can be affected by the accumulation of advanced glycation end products and low-grade inflammation, which can be underdiagnosed as a result of the delay in presenting symptoms and the large pulmonary physiological reserve [53]. A study proves that glycemic exposure is a strong determinant of reduced lung function in T2DM, and the decline in lung capacity is faster in patients with higher baseline HbA1c, consequently, poor glycemic control [55].

Therefore, medications that decrease the inflammation seem to be the key to controlling asthma when associated with proinflammatory conditions such as T2DM [37, 38]. Thus, it is essential to mention studies carried out with other drugs such as GLP-1 analogs, which have shown a significant decrease in the inflammatory process, placing those medications as possible candidates for additional treatment in the treatment of people with concomitant asthma and T2DM, once that incretins are able to act in glycemic control (increase glucose-dependent insulin secretion and inhibit glucagon secretion and hepatic glucose production) and act in the control of obesity and metabolic syndrome (decrease gastric emptying, act on the mechanisms of appetite and satiety at the central level and on adiposity), in addition to exercising direct and indirect actions on the cardiovascular system, modulating the inflammatory activity [66, 67].

Finally, although there is little evidence showing the direct impact of T2DM on asthma control, studies show that patients with metabolic disorders such as obesity and MS have poor control of this chronic condition. Barros et al. observed that patients with severe asthma and class III obesity were more likely to have uncontrolled asthma than those with normal BMI [68]. A study carried out in Nigeria, comparing asthma control in individuals with and without MS, demonstrated a worse symptom control in those with MS when compared to their peers without the metabolic disorder [7]. The study by Forno et al. with 433 obese and asthmatic patients with MS undergoing bariatric surgery unveiled that having MS increased the chance of losing control of asthma during follow-up (OR = 1.92), negatively modifying the effect of weight loss on the control of induced asthma for this treatment [69].

## 5. Final Thoughts

Asthma and T2DM seem to coexist in the same patient. In particular, low-grade systemic inflammation and the use of medications such as corticosteroids may be behind this association. This association seems to favor the lack of glycemic control and greater impairment of lung function, worsening asthma control.

It is essential to expand the investigation of asthma comorbidities, given the growing number of studies that suggest low-grade systemic inflammation as a common pathophysiological basis. Other pathways that affect the prognosis of asthma and are involved in increasing the risk for the development of other chronic conditions that also have high morbidity should be identified, which may pave the way for new therapeutic and preventive possibilities, with a positive impact on the quality of life of individuals and for the public health.

## Figures and Tables

**Table 1:** Description of the articles, 2019.

Source	Study site	Sample characteristics	Study design	Results
Gulcan et al. [30]	Kutahya/Peru	91 participants (51 asthma patients and 40 nonasthmatic controls). Participants with asthma: $\overset{⌣}{x}$ = 40.3 ± 7.8; nonasthmatic controls: $\overset{⌣}{x}$ = 39.5 ± 6.7.	Cross-sectional design	Changes in fasting blood glucose levels and 2 hours after coffee, plasma insulin, HOMA-IR and LDL-C levels were greater in patients with asthma than in nonasthma controls (*p* < 0.005).

Hashemzadeh and Movahed, [29]	Arizona/USA	845,748 participants (293,124 with diabetes and without hypertension and 552,625 with hypertension and without diabetes as controls); asthma and diabetes: 13,243; asthma and hypertension: 16,036. Patients with T2DM $\overset{⌣}{x}$ = 65.8 ± 11.3 and the control group NON-T2DM $\overset{⌣}{x}$ = 64.8 ± 12.6; men: 97.8% in the T2DM group and 97.4% in the control group.	Cross-sectional design	Asthma was present in 4.5% of patients with T2DM and 2.9% in the control group. After adjusting for covariables such as hyperlipidemia and smoking, T2DM remained independently associated with asthma (OR = 2.99; 95% CI: (2.92–3.06); *p* < 0.001).

Faul et al. [34]	California/USA	10 male adults with DMT2 and asthma or COPD (05 with asthma and 05 with COPD). All participants: $\overset{⌣}{x}$ = 64 ± 52–76. All male.	Prospective, randomized, double-blind, double-placebo, placebo-controlled study	The mean of HbA1c after 06 weeks of treatment with fluticasone vs. treatment with oral montelukast was significantly higher than the mean of the baseline but was not considered clinically relevant (mean differences = 0.11 and −0.14, respectively, *p*=0.025).

Song et al. [15]	Boston/USA	38,570 women (3,368 asthma only; 1,808 COPD only; 32,248 controls-no). Mean age of asthma only: 53.8.	Prospective cohort	Women who have ever reported asthma were associated with an increased risk of diabetes: RR = 1.37 (95% CI: [1.20–1.57]). This association was not significantly modified by confounders such as age, smoking, or BMI.

Suissa et al. [33]	Quebec/Canada	358,417 participants treated for respiratory disease (30,167 had diabetes-incidence rate: 14.2/100/year) and 2,099 progressed from oral hypoglycemic to insulin-incidence rate: 19.8/1000/year). Participants diagnosed with diabetes: $\overset{⌣}{x}$ = 66.3 ± 15 (cases and control); participants with progression to diabetes: $\overset{⌣}{x}$ = 65.5 ± 16.0 (cases) and $\overset{⌣}{x}$ = 65.4 ± 15.6 (controls); male with diabetes: 41.4% (cases) and 38.7 (controls); progressed to insulin = 37.6% (cases) and 37.5% (controls).	Prospective cohort	The use of CIS was associated with a 34% increase in the incidence of diabetes (RR = 1.34; 95% CI: 1.29–1.39) and a 34% progression rate of the oral hypoglycemic to insulin (RR = 1.34; 95% CI: [1.17–1.53]); the risks were higher with the higher doses of CIS, equivalent to fluticasone 1000 *µ*g/day or more (RR = 1.64; 95% CI: (1.52–1.76) and RR = 1.54; 95% CI: (1.18–2.02), respectively)

Thomsen et al. [28]	Copenhagen/Denmark	34,782 Danish twins (3,004 with asthma and 31,778 nonasthma controls). Average age: 20–71 years, being 54% women and 46% men.	Cross-sectional design	The risk of asthma was increased in individuals with T2DM compared to nondiabetic individuals, both in men (13.5% vs. 7.5%; *p*=0.001) and in women (16.6% vs. 9.6%; *p*=0.001).

Yun et al. [19]	Rochester, Minnesota/USA	7,176 participants (asthma = 2,392 and nonasthma controls = 4,784). Age at onset of asthma: $\overset{⌣}{x}$ = 15.1 ± 20.5; adults ≥18 years: 1,310 (55%) asthma group; 2,690 (56%) control group; male = 1,356 (57%) asthma group; 2,712 (57%) controls.	Retrospective cohort	Asthma is associated with an increased risk of diabetes (OR = 2.11; 95% CI: (1.43–3.13); *p* < 0.001).

Rodbard et al. [27]	Maryland/USA	8,582 baseline participants: 7,970 non-DM2 and 622 transitioned to T2DM. Non-T2DM participants: $\overset{⌣}{x}$ = 53.7 ± 16.5; made the transition to T2DM: $\overset{⌣}{x}$ = 58.3 ± 12.5; non-T2DM women = 61.5% and women transitioning to T2DM = 61.3%.	Prospective cohort	Self-reported asthma was associated with an increased risk of transition to T2DM (OR = 1.33; 95% CI: [1.04–1.70]; *p*=0.020).

Koskela et al. [32]	Kuopio/Finland	153 patients (109 with asthma and 44 with COPD); 23 with previous diabetes diagnosis and 103 without diabetes diagnosis; without diabetes: 103 with hyperglycemia and 27 with euglycemia. Participants with diabetes: 56% male and $\overset{⌣}{x}$ = 65.0 ± 12.9; participants without diabetes with hyperglycemia: 55% male and $\overset{⌣}{x}$ = 65.9 ± 15.0.	Cross-sectional design	Of the entire study population, 125 (82%) demonstrated hyperglycemia: 103 (79%) among patients without diabetes and 22 (96%) among patients previously diagnosed with diabetes. The prevalence of hyperglycemia did not differ among patients with asthma and COPD.

Mueller et al. [14]	Republic of Singapore/Southwest Asia	42,842 men and women (873 with asthma and 41,969 nonasthma controls). Men and women: nonasthma $\overset{⌣}{x}$ = 55.2 ± 7.6; asthma >18 years: $\overset{⌣}{x}$ = 56.7 ± 8.1; asthma <18 years: $\overset{⌣}{x}$ = 52.5 ± 6.5.	Prospective cohort	Asthma was associated with a 31% increased risk of diabetes incidence (OR = 1.31; 95% CI: [1.00–1.72]). The association was attenuated after adjusting for BMI (OR = 1.25; 95% CI: [0.95–1.64]).

Koskela et al. [39]	Kuopio/Finland	153 participants: 110 (72%) with asthma and 43 (28%) with COPD; 23 with a medical diagnosis of diabetes; 20 diagnosed during screening; 110 without diabetes. Diabetes diagnosis: $\overset{⌣}{x}$ = 64.4 (59.1–69.9), 52% male; diabetes screening: $\overset{⌣}{x}$ = 68.8 (64.8–72.8), 70% male; nondiabetes: $\overset{⌣}{x}$ = 64.3 (61.3–67.2), 51% male.	Prospective cohort	Fasting hyperglycemia was detected in 91% of patients diagnosed with diabetes, 90% of patients screened for diabetes, and 63% of nondiabetes (*p* = 0.003). Previously diagnosed diabetes was associated with high mortality (aOR = 1.04; 95% CI: (0.50-A 2.12).

Wytrychowsky et al. [20]	Wroclaw/Poland	88 patients hospitalized for acute asthma exacerbation (24 with glucose disorders and 64 controls without hyperglycemia). Group *A* = 11 patients/intravenous insulin: $\overset{⌣}{x}$ = 61.4 ± 11.0; group *B* = 13 patients/ subcutaneous insulin; $\overset{⌣}{x}$ = 53.7 ± 12.5; group *C* = 64 patients without hyperglycemia/control group: $\overset{⌣}{x}$ = 48.3 ± 14.4.	Prospective, randomized study	Newly diagnosed hyperglycemia and/or diabetes hinders recovering from acute asthma exacerbation requiring hospitalization, prolonging hospital stay compared to patients who do not have glucose metabolism disorders.

Sweeney et al. [23]	Belfast/United Kingdom	7,195 participants (severe asthma: 808; mild/moderate asthma: 3,975; nonasthma control: 2,412). All participants: $\overset{⌣}{x}$ = 58 ± 17; severe asthma: $\overset{⌣}{x}$ = 58 ± 18; mild/moderate asthma: $\overset{⌣}{x}$ = 58 ± 16; nonasthma control: $\overset{⌣}{x}$ = 58 ± 17.	Cross-sectional design	In comparison with mild/moderate asthma, patients with severe asthma had higher morbidity rates when exposed to systemic corticosteroids (T2DM: 10% vs. 7%, OR = 1.46; 95% CI [1.39–2.56; *p* < 0.001]).

Verbovoy et al. [31]	Samara/Russia	53 women (27 with a diagnosis of DM2; 26 with a combination of T2DM and asthma; 23 from the control group). Women diagnosed with T2DM ($\overset{⌣}{x}$ = 60.15 ± 0.92 years); combination of T2DM and asthma ($\overset{⌣}{x}$ = 61.23 ± 0.95 years); control group (**x** = 51.26 ± 1.73).	Cross-sectional design	Hyperleptinemia, hyperresistinemia, lower adiponectin concentrations, higher levels of proinflammatory IL-6 and anti-inflammatory IL-10, and significantly elevated blood glucose levels were found in patients with T2DM and asthma than in the control group. These changes occurred in the presence of subclinical inflammation.

Klein et al. [21]	California/USA	2,645 men and women (659 with lung disease (asthma, bronchitis, and COPD) and T2DM; 1,986 with lung disease and non-T2DM). Participants with pulmonary disease and T2DM: $\overset{⌣}{x}$ = 53.8 (52.3–55.2) and with lung and non-T2DM disease: $\overset{⌣}{x}$ = 39.3 (38.4–40.2; women with lung disease and T2DM: $\overset{⌣}{x}$ = 63.4 (61.9–64.8) and with lung disease and non-T2DM: $\overset{⌣}{x}$ = 58.2 (55.8–60.6).	Multicenter and observational study with cross-sectional analyses	Among Hispanics/Latinos with lung disease, those with T2DM have lower mean FEV1 and FVC values than those without T2DM (VEF1 3,00; IC: [2,96–3,04] vs. 3,10; IC95%: [3,09–3,11], *p* < 0.01) *e* CVF (3,62; 95% IC: [3,59–3,66] vs. 3,81; 95% IC: [3,79–3,83], *p* < 0.001).

Li et al. [38]	Taipei/Taiwan	1,332 patients with asthma and diabetes (444/33.3% using metformin and 888/66.6% non-metformin). Participants using metformin: 64 (10.4%); female: 268 (60.4%); male: 176 (39.6%); non-metformin: 64 (10.4%); female: 536 (60.4%); male: 352 (39.6%).	Retrospective cohort	In comparison with nonusers, metformin users had a lower risk of hospitalization related to asthma (OR = 0.21, 95% CI: [0.07–0.63]) and exacerbation of asthma (OR = 0.39, 95% CI: [0.19–0.79]).

Veryomenko and Bezditko [37]	Karkov/Ukraine	47 patients with asthma and T2DM before and after treatment. Treatment (group 1, *n* = 28) and comparison (group 2, *n* = 19). Data not shown.	Intervention study	The use of the L-arginine complex and TB preparations against the use of basic therapy in patients with asthma and T2DM produced better control over the disease, faster elimination of manifestations of obstruction, clinical spirographic remission, improved quality of life, correction of disorders in hemocoagulation, fibrinolysis, and functional status of the endothelium.

Baek et al. [16]	Korea	13,154,348 (2,335,303 with asthma and 10,819,045 controls without asthma). Nonasthma: $\overset{⌣}{x}$ = 48 ± 12.3; asthma: $\overset{⌣}{x}$ = 52 ± 13.1.	Cross-sectional design	Diabetic patients with retinopathy had an increased risk of developing asthma (OR: 1.067; 95% CI: [1.053–1.081]). Diabetic patients without retinopathy had a lower risk of developing asthma compared to nondiabetics (OR: 0.943; 95% CI: [0.939–0.948]).

$\overset{⌣}{x}$: mean; ±: standard deviation; HOMA IR: homeostasis model assessment-insulin resistance; LDL-C: low density lipoprotein cholesterol; OR: odds ratio; aOR: odds ratio adjusted; CI: confidence interval; HbA1c: glycated hemoglobin; RR: relative risk; BMI: body mass index; T2DM: diabetes mellitus type 2; CIS: inhaled corticosteroid; COPD: chronic obstructive pulmonary disease; IL: interleukin; FEV1: forced expiratory volume in the first minute; FVC: forced vital capacity; TB: tiotropium bromide.

## Data Availability

The data that support this are from previously reported studies and data sets, which were cited. The processed data are available from the corresponding author upon request.
